# Genome-wide transcriptome analysis of porcine epidemic diarrhea virus virulent or avirulent strain-infected porcine small intestinal epithelial cells

**DOI:** 10.1016/j.virs.2022.01.011

**Published:** 2022-01-18

**Authors:** Ouyang Peng, Xiaona Wei, Usama Ashraf, Fangyu Hu, Yongbo Xia, Qiuping Xu, Guangli Hu, Chunyi Xue, Yongchang Cao, Hao Zhang

**Affiliations:** a State Key Laboratory of Biocontrol, Life Sciences School, Sun Yat-sen University, Guangzhou, 510006, China; b Wen's Group Academy, Wen's Foodstuffs Group Co, Ltd, Xinxing, Guangdong, 527400, China; c State Key Laboratory of Agricultural Microbiology, Huazhong Agricultural University, Wuhan, 430070, China; d Guangdong Provincial Key Laboratory of Malignant Tumor Epigenetics and Gene Regulation, Sun Yat-sen Memorial Hospital, Sun Yat-sen University, Guangzhou, 510120, China

**Keywords:** Coronavirus, Porcine epidemic diarrhea virus (PEDV), Virulence, Transcriptome, Apoptosis, Autophagy, Immunity

## Abstract

Porcine epidemic diarrhea virus (PEDV) is the main cause of diarrhea, vomiting, and mortality in pigs, which results in devastating economic loss to the pig industry around the globe. In recent years, the advent of RNA-sequencing technologies has led to delineate host responses at late stages of PEDV infection; however, the comparative analysis of host responses to early-stage infection of virulent and avirulent PEDV strains is currently unknown. Here, using the BGI DNBSEQ RNA-sequencing, we performed global gene expression profiles of pig intestinal epithelial cells infected with virulent (GDS01) or avirulent (HX) PEDV strains for 3, 6, and 12 ​h. It was observed that over half of all significantly dysregulated genes in both infection groups exhibited a down-regulated expression pattern. Functional enrichment analyses indicated that the differentially expressed genes (DEGs) in the GDS01 group were predominantly related to autophagy and apoptosis, whereas the genes showing the differential expression in the HX group were strongly enriched in immune responses/inflammation. Among the DEGs, the functional association of TLR3 and IFIT2 genes with the HX and GDS01 strains replication was experimentally validated by TLR3 inhibition and IFIT2 overexpression systems in cultured cells. TLR3 expression was found to inhibit HX strain, but not GDS01 strain, replication by enhancing the IFIT2 expression in infected cells. In conclusion, our study highlights similarities and differences in gene expression patterns and cellular processes/pathways altered at the early-stage infection of PEDV virulent and avirulent strains. These findings may provide a foundation for establishing novel therapies to control PEDV infection.

## Highlights

• Differentially expressed genes (DEGs) in the GDS01 group were predominantly related to autophagy and apoptosis.
• DEGs in the HX group were strongly enriched in immune responses/inflammation.
• TLR3 inhibits HX strain replication, but not GDS01 strain, by enhancing the IFIT2 expression.

## 1 Introduction

Coronaviruses (CoVs) are enveloped, positive-sense, single-stranded, RNA viruses that are known to infect a variety of mammalian species, such as humans, pigs, bats, and camels (Haagmans et al., 2014; Niederwerder et al., 2018; Wong et al., 2007; Zhou et al., 2020). CoVs, belonging to the family *Coronaviridae*, are classified into four genera: *Alphacoronavirus*, *Betacoronavirus*, *Gammacoronavirus*, and *Deltacoronavirus* (Cui et al., 2019; Woo et al., 2012). Of these, the viruses belonging to the genus *Betacoronavirus* can jump from animals to humans, and thus, can be responsible for lethal acute respiratory illnesses in humans such as the Middle East respiratory syndrome and the severe acute respiratory syndrome (Drosten et al., 2003; Zaki et al., 2012). Notably, the recent pandemic of Coronavirus Disease 2019, caused by the severe acute respiratory syndrome-CoV2, infected over 80 million people with exceeding 1.8 million deaths globally (Sun et al., 2020; WHO, 2021; Wu et al., 2020). Moreover, the bat-HKU2-like novel porcine CoV has been identified to cause acute diarrhea syndrome in pigs (Gong et al., 2017; Zhou et al., 2018). The replication of bat-HKU2-like CoV in multiple mammalian cells highlighted its ability to cross the species barrier (Edwards et al., 2020), which requires great attention.

Porcine epidemic diarrhea virus (PEDV), belonging to the genus *Alphacoronavirus*, is the causative agent of porcine epidemic diarrhea (PED) disease, and it has been a considerable threat to pigs of all ages with a mortality rate of ∼ 100% in suckling piglets caused by virulent strains (Li et al., 2012). PED is a highly devastating enteric disease which is characterized by vomiting, diarrhea, dehydration, weight loss, and eventually death (Madson et al., 2014). Since its first isolation in the United Kingdom and Belgium in the 1970s, PEDV has been reported in several countries, including the United States, Canada, Mexico, China, Korea, and Japan, resulting in serious damage to the pig industry and huge economic losses (Chen et al., 2012; Chung et al., 2015; Mole, 2013; Niederwerder et al., 2018; Takahashi et al., 1983). PEDV primarily infects the cells of the small intestine, and pig-to-pig transmission mainly occurs by fecal-oral route (Li et al., 2018). PED outbreaks were found to be associated with the novel virulent recombinant PEDV strains (G2 subgroup), which were genetically distinct from the classic avirulent CV777 strain (G1 subgroup) (Chen et al., 2014; Huang et al., 2013; Wang et al., 2020).

High-throughput transcriptomic approaches are extensively used for studying the molecular forces deriving the virus-host systems. The RNA-sequencing analysis of the PEDV-infected cultured pig intestinal cells revealed the perturbation of signal transducer and activator of transcription, cell cycle, and apoptosis pathways at the later stages of infection (Shen et al., 2020). In a similar context, a few studies have identified several key host genes involved in regulating the antiviral immune response against PEDV such as FOSL1, IL1A, ISG15, and some other ISGs (Hu et al., 2020; Sun et al., 2019; Wang et al., 2019). However, the host transcriptomic landscape at an early phase of PEDV infection and differences in the host response upon infection with an avirulent or a virulent PEDV strain are currently unknown.

In this study, using the DNBSEQ RNA-sequencing, we comprehensively analyzed the genome-wide transcriptomic profiles of cellular mRNAs isolated from cultured porcine small intestinal epithelial cells (IPEC-J2 cells) infected with virulent (GDS01) or avirulent (HX) PEDV strains at three points of early stages of virus infection. Our data identified PEDV-induced widespread alterations in cellular mRNA expression and highlighted key differences in the host response to a virulent and an avirulent PEDV strain. This study may shed light on the biology of PEDV infection that can be considered for developing antiviral therapies against PEDV.

## 2 Materials and methods

### 2.1 Cells and viruses

Porcine intestinal columnar epithelial cells (IPEC-J2 cell line) were grown in Dulbecco's modified Eagle's medium (#11995, Gibco, New York NY, USA) containing 5% fetal bovine serum (#10100, Gibco), 100 U/mL penicillin, and 100 ​mg/mL streptomycin. PEDV strains HX (avirulent, accession number: MH726408) and GDS01 (virulent, accession number: KM089829), belonging to the subgroup G1 and G2, respectively, were isolated and propagated in our laboratory, which were described by our previous study (Wei et al., 2020). The median tissue culture infectious dose (TCID_50_) of both viral strains were as follows: HX strain, 1.47 ​× ​10^−5^/0.1 ​mL and GDS01 strain, 2.15 ​× ​10^−6^/0.1 ​mL.

### 2.2 Samples preparation and RNA extraction

**Fig. 1:**
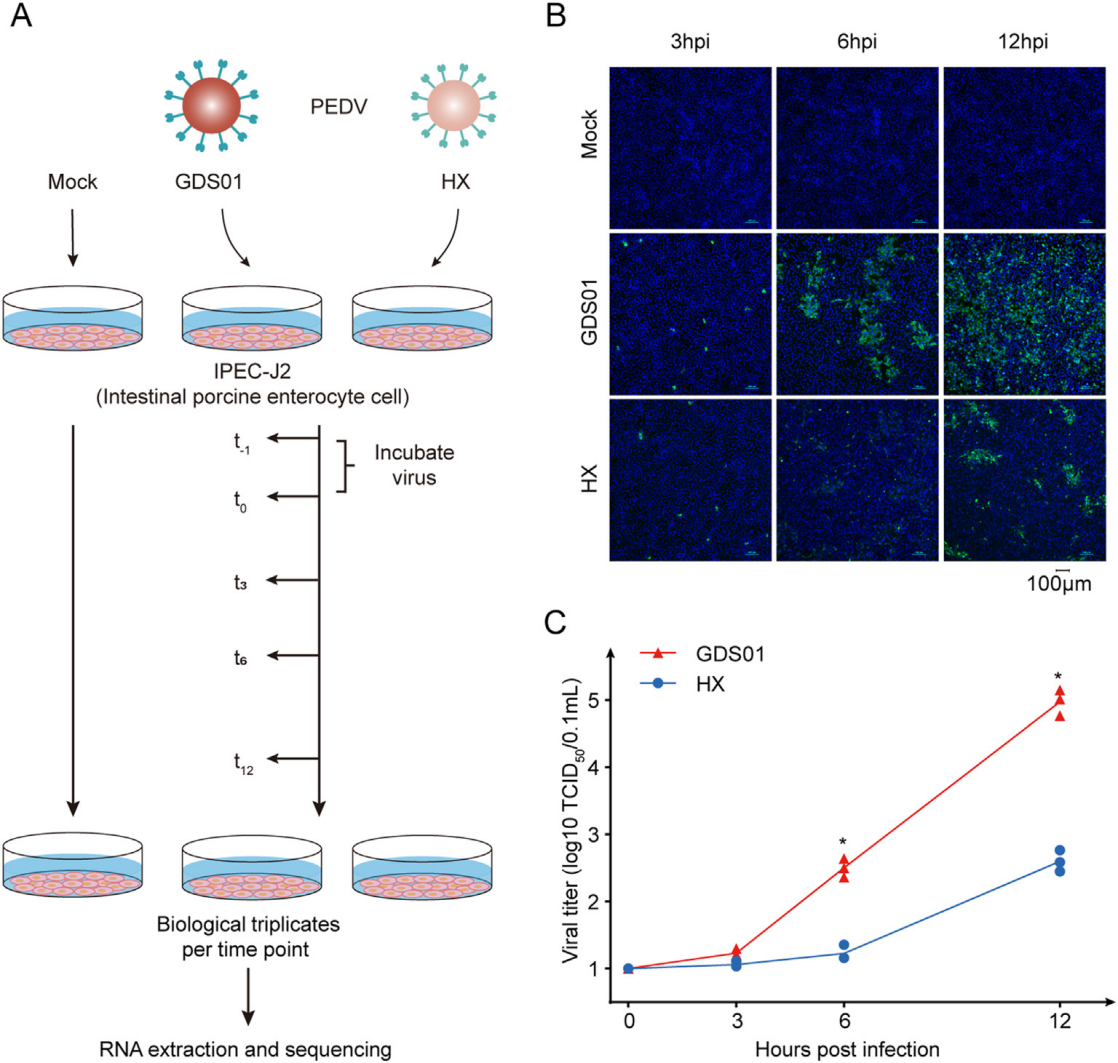
Workflow and confirmation of productive PEDV infection in cultured IPEC-J2 cells. **A** Workflow. IPEC-J2 cells were mock-infected or infected with PEDV strain GDS01 or HX (MOI ​= ​1), followed by sample collection at 3, 6, 12 hpi. Samples from each group were prepared in triplicate. Total RNA was extracted and BGI DNBSEQ RNA-sequencing was performed. **B** Immunofluorescence staining of the viral S-protein in PEDV-infected IPEC-J2 cells. Cells were fixed at 3, 6, or 12 hpi, and S protein (green) was detected by indirect immunofluorescence assay. Nuclei (blue) were shown by 4′,6-diamidino-2-phenylindole (DAPI) staining. The images of cells were acquired by a fluorescence microscope (Nikon Eclipse 80i) at a 20× magnification. **C** Viral titers in the culture supernatants were determined by TCID_50_ assay. Data are shown as mean ​± ​SEM and representative of three independent experiments. Data were analyzed by the Mann-Whitney test. ∗*P* ​< ​0.05. PEDV, porcine epidemic diarrhea virus; MOI, multiplicity of infection; SEM, standard error of mean.

IPEC-J2 cells plated in six-well plates (1 ​× ​10^6^ ​cells/well) were infected with PEDV strain GDS01 or HX at an MOI of 1 for a period of 0, 3, 6, and 12 ​h. After infection, cells were washed with 1 ​× ​phosphate-buffered saline (PBS) followed by total RNA extraction using the TRIzol reagent (#15596, Thermo Fisher Scientific, Waltham MA, USA). Samples harvested at 0 ​h post-infection (hpi) were considered as mock-infected. Three biological replicates were set per time point (Fig. 1A).

### 2.3 Library preparation and RNA-sequencing

The mRNA of samples was purified by magnetic beads connected with oligo(dT) and were then fragmented into small pieces with the fragment buffer. Random hexamer-induced reverse transcription was used to generate first-strand cDNA, followed by second-strand cDNA synthesis. Afterward, the A-tailing mixture and RNA Index Adapters were added by incubation to end repair. The cDNA fragments obtained from the previous step were amplified by PCR, and products were purified by Ampure XP beads, and then dissolved into the EB solution. The products were evaluated using the Agilent Bioanalyzer 2100 (Agilent Technologies, Santa Clara CA, USA). The double-stranded PCR products from the previous step were heated, denatured, and circularized by the splint oligo sequence to obtain the final library. The final library was then amplified with phi29 to prepare DNA nanoball (DNB) with more than 300 copies of one molecule. DNBs were loaded into the patterned nanoarray and single-ended 150 base reads were generated on the BGIseq500 platform (BGI, Wuhan, China).

### 2.4 Sequencing data quality control and genome mapping

The quality control of raw reads was performed using the SOAPnuke software (Cock et al., 2010). The following reads were filtered out: 1) the reads containing adaptors, 2) the reads with N content more than 5%, and 3) low-quality reads (reads with a base quality score of less than 10 accounted for more than 20% of the total bases as low-quality reads). The filtered clean reads obtained from each sample were ∼ 45.83 million, packaged in the form of FASTQ, with an average size of 6.87 ​Gb. The Hierarchical Indexing for Spliced Alignment of Transcripts (HISAT) was used to map RNA-sequencing reads to the porcine reference genome (RefSeq assembly accession: GCF_000003025.6). Firstly, we use HISAT global FM index to anchor the position of partial sequences in each read on the genome, and then, use the partial genome indexes of these alignment positions to align the remaining sequences of each read to extend the alignment area (Kim et al., 2015).

### 2.5 Gene expression analysis

In order to quantitatively analyze the gene expression level, Bowtie2 (V2.2.5) was employed to align the clean reads to the porcine reference gene sequences (Langmead et al., 2012). The soft parameters were set as: -q --phred64 --sensitive --dpad 0 --gbar 99999999 --mp 1,1 --np 1 --score-min L, 0, −0.1 -p 16 -k 200. And then, the RSEM (V1.2.8) was used to calculate and normalize the gene expression level in each sample with the default parameters (Li et al., 2011). Differentially expressed genes (DEGs) were selected by applying the following filtering parameters: |log2 (fold change)| ​≥ ​1, and Q-value ≤ 0.01.

### 2.6 Gene ontology (GO) term and pathway enrichment analysis of DEGs

GO term and pathways enrichment analysis were performed respectively using GO::TermFinder (Boyle et al., 2004) and Kyoto encyclopedia of genes and genomes (KEGG) pathways database (Kanehisa et al., 2008). For GO term analysis, we mapped all DEGs to each term in the GO database (Harris et al., 2004), calculated the number of genes in each term, and then applied the hypergeometric test to find GO terms that were significantly enriched in candidate genes compared with the background of all genes of the pig. The calculated *P*-value was corrected by Bonferroni. The calculation method for predicting enriched pathways was the same as that described for the GO terms. The GO term or pathway with Q-value (corrected *P*-value) ​≤ ​0.05 was defined as a significantly enriched GO term or pathway corresponding to the candidate gene.

### 2.7 Quantitative real-time PCR

To analyze mRNA expression, 0.1 ​μg of total RNA was used to reverse transcribe in order to construct cDNA using the First Strand cDNA Synthesis Kit (#FSK-101, TOYOBO, Tokyo, Japan) containing oligo(dT) primer (5′-TTTTTTTTTTTTTTTTTTTT-3′) according the manufacturer's protocol. Quantitative real-time PCR was performed using the SYBR Green Real-time PCR Master Mix (#11201ES03, Yeasen, Shanghai, China) and a LightCycler480 II system (Roche, Basel, Switzerland). Amplification was performed at 50 ​°C for 2 ​min, 95 ​°C for 10 ​min, and then followed by 40 cycles of 95 ​°C for 15 ​s, 60 ​°C for 15 ​s, and 72 ​°C for 30 ​s. The relative expression values of candidate mRNAs were normalized to that of GAPDH in each sample using the 2^−ΔΔCt^ method.

### 2.8 Quantification of virus copies

The number of PEDV genomic copies was assessed with a SYBR Green Real-time PCR Master Mix (Yeasen) and a LightCycler480 II system (Roche). The qRT-PCR conditions used were a holding stage of two steps, which included a first step at 48 ​°C for 30 ​min, and a second at 95 ​°C for 10 ​min. The cycling stage included 40 repeated cycles of two steps, a first step of 15 ​s at 95 ​°C and a second step of 1 ​min at 60 ​°C. The melt curve stage was composed of four steps, a first step of 1 ​min at 95 ​°C, a second step of 1 ​min at 60 ​°C, a third step with a gradual increase in temperature with 0.35 ​°C for 0.3 ​s to obtain a temperature of 95 ​°C and a fourth step of 15 ​s at 60 ​°C. The primers used for the PCR were designed from the conserved regions of the PEDV nucleocapsid gene for universal detection of the strain used for inoculation (forward, 5′-CGCAAAGACTGAACCCACTAA-3′; reverse, 5′-TTGCCTCTGTTGTTACTTGGAGAT-3′).

### 2.9 TLR3 inhibition assay

IPEC cells were seeded in 6-well plates at a density of 1 ​× ​10^6^ ​cells/well and grew for 24 ​h. Non-adherent cells and media were removed and replaced with fresh DMEM, and adherent IPEC cells were then treated with commercially available selective TLR3 inhibitor CU CPT 4a (#4883, R&D, Minneapolis MN, USA) at IC_50_ ​= ​3.44 ​μM for a period of 1 ​h. Later, treated cells were mock-infected or infected with GDS01 or HX strain (MOI ​= ​1) for a period of 3, 6, and 12 ​h. Subsequently, samples were collected and processed for further experiments.

### 2.10 Plasmid construction and IFIT2 overexpression assay

For the construction of the pCMV-Flag-IFIT2 plasmid, the cDNA derived from IPEC cells was utilized as a template to amplify the porcine IFIT2 gene by PCR, followed by cloning into the pCMV-Flag vector (Supplementary Fig. S3). IPEC cells were seeded in 6-well plates at a density of 1 ​× ​10^6^ ​cells/well and grew for 24 ​h. Cultured cells were either transfected with empty vector or pCMV-Flag-IFIT2 (2 ​μg each) using the Lipofectamine 3000 (#L3000008, Thermo Fisher Scientific). At 36 ​h post-transfection, the cells were infected with GDS01 or HX strain (MOI ​= ​1) for a period of 3, 6, and 12 ​h. Subsequently, samples were collected and processed for further experiments.

### 2.11 Poly(I:C) transfection

IPEC cells were seeded in 6-well plates as described above and incubated for 24 ​h. Then the culture medium was removed and polyinosinic-polycytidylic acid [Poly(I:C)] was transfected at 0.5 ​mg/mL using Lipofectamine 3000. At 12 ​h post-transfection, cells were infected with GDS01 or HX strain at an MOI of 1 for 3, 6, and 12 ​h. Subsequently, the cell supernatants were collected and processed for the quantification of viral copies and titers by quantitative real-time PCR and TCID_50_ assay, respectively.

## 3 Results

### 3.1 IPEC-J2 cells are permissive to infection caused by PEDV GDS01 and HX strains

To investigate the dynamics of host genes expression during the early stages of the PEDV infection, cultured IPEC-J2 cells were mock-infected or infected with either the PEDV GDS01 strain (virulent) or PEDV HX strain (avirulent) at MOI of 1 for a period of 3, 6, and 12 ​h (Fig. 1A). To validate whether IPEC-J2 cells have acquired successful infection, the replication of GDS01 and HX viruses was examined by an immunofluorescence assay (IFA) and the TCID_50_ assay. It was observed that both viral strains replicated successfully in a time-dependent manner as assessed by the increasing number of PEDV S protein-positive cells (Fig. 1B) and the increasing production of infectious viral particles (Fig. 1C). In both assays, the GDS01 strain was observed to reach at significantly higher virus titers compared to the HX strain at 6 ​h and 12 ​h infection time-points (Fig. 1B and C). Overall, these results indicate the establishment of PEDV GDS01 and HX strains infection in cultured IPEC-J2 cells.

### 3.2 Transcriptomic analysis of the PEDV GDS01 or HX strain-infected IPEC-J2 cells

**Fig. 2:**
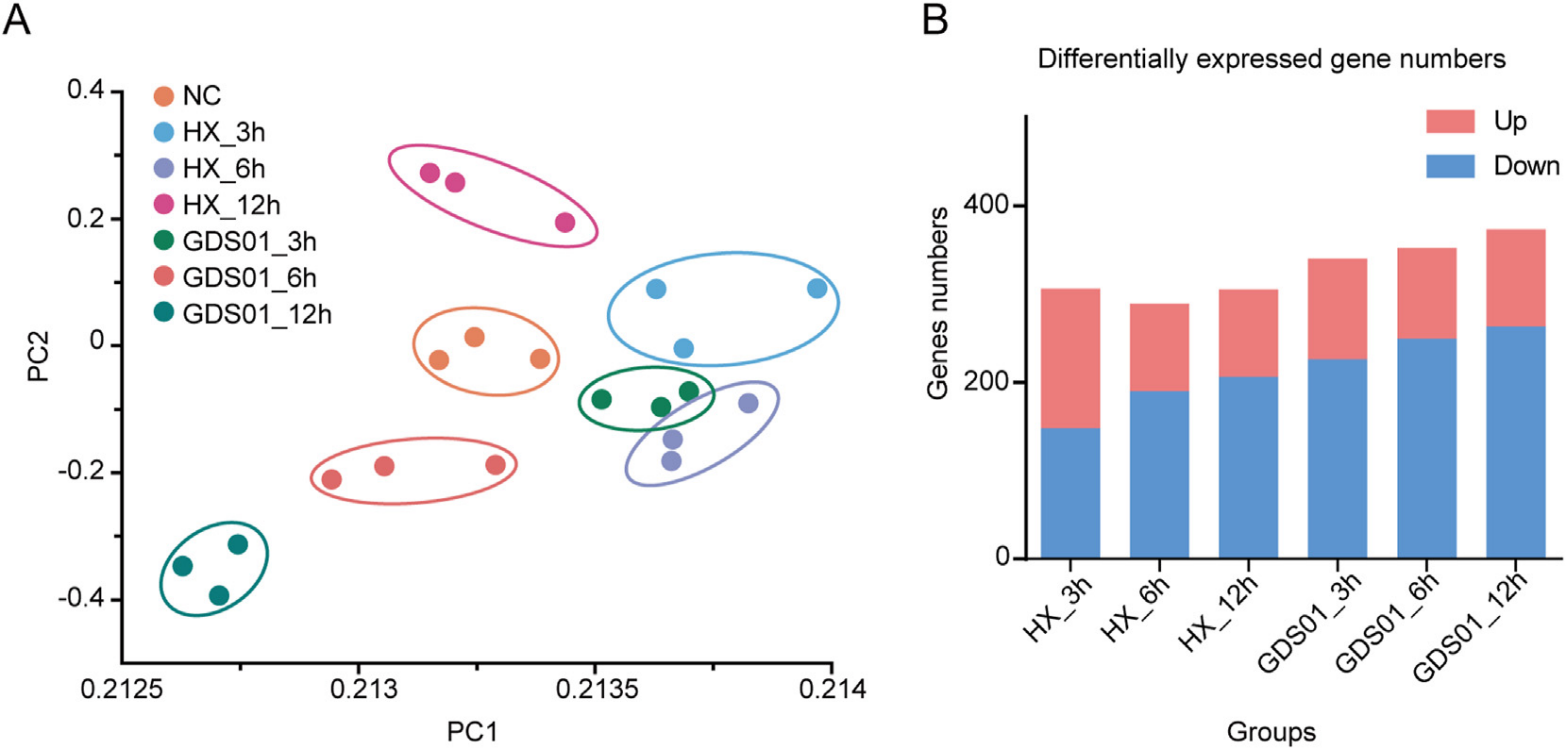
RNA-seq analysis of PEDV strains-infected IPEC-J2 cells. **A** PCA analysis. The principal component of each sample was analyzed considering the gene expression in the corresponding sample. Samples corresponding to each experimental group (three biological replicates per group) were plotted on the first two principal components. **B** Barplot demonstrating the abundance of differentially expressed genes in PEDV GDS01 or HX-infected IPEC-J2 cells compared with mock-infected cells at each time point. PEDV, porcine epidemic diarrhea virus; PCA, principal component analysis.

To examine the reproducibility and the specificity of each group, the gene expression levels were used to conduct a principal component analysis (PCA) for each biological replicate. Every sample from the same group was clustered closely together, which suggested that the reproducibility of each treatment was satisfactory, and the specificity between groups was apparent (Fig. 2A).

In order to visualize the transcriptomic profile of the GDS01- or HX -infected cells, a total of 21 sequencing libraries prepared at 0, 3, 6, and 12 hpi were sequenced, and ∼ 976 million raw reads were obtained. The number of clean reads ranged from ∼ 44 to 48 million after filtering out the adapter and low-quality reads. To this end, a total of 1965 significant DEGs were identified in infected samples when compared to mock infected samples under the parameter of Q-value ≤ 0.01 and |log2 (fold change)| ​≥ ​1. Overall, approximately half of the genes in HX-infected groups at 3 hpi are down regulated, whereas genes in other groups exhibiting the down-regulated expression pattern constitute two third of total DEGs identified, and the increase in the number of down-regulated genes is dependent on the time of infection (Fig. 2B). Moreover, the total number of DEGs detected in all three infection time-points is more in the GDS01-infected group compared to the HX-infected group (Fig. 2B), which may associate with the differences in the replication of both viral strains as observed in Fig. 1B and C. Taken together, these data demonstrate that the infection of cultured IPEC-J2 cells by PEDV GDS01 or HX strain induces widespread alterations in the expression pattern of host genes.

Venn diagrams were generated to examine the unique and overlapping DEGs among subgroups with the same virus strain infection at three infection time-points or among subgroups with different strains infection but the same infection time-point. To this end, 55 and 38 DEGs were noticed common among all three subgroups in the GDS01 and HX groups, respectively. However, no DEG was found common among all three infection time-points belong to both infection groups, suggesting a marked differential host response at each stage of early infection (Supplementary Fig. S1B). In the HX subgroups, immune-associated DEGs, such as GATA3, FOS, IFIR2, IL1R2, CXCL10, were detected at each time-point, whereas in the GDS01 subgroups, only IL1R2 and CXCL10 genes were identified.

### 3.3 Weighted gene co-expression networks analysis

**Fig. 3:**
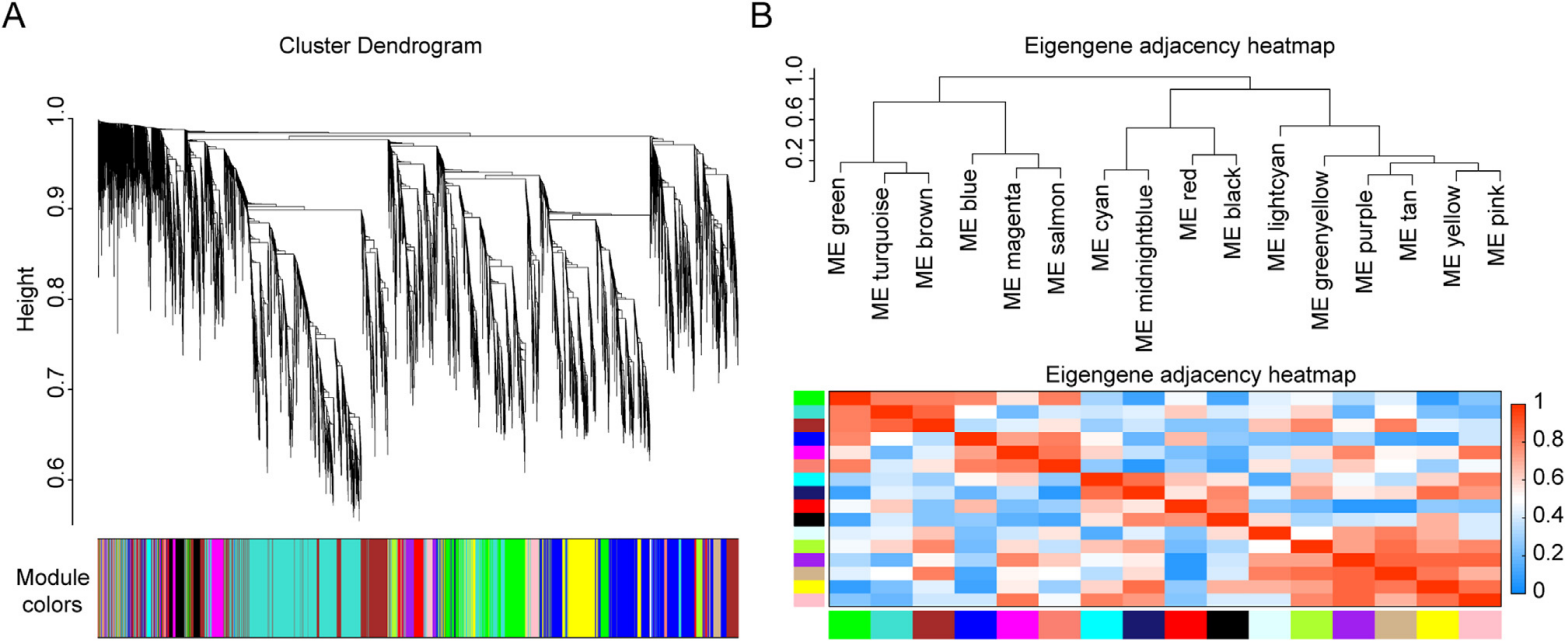
Construction of a weighted gene co-expression network. **A** A cluster dendrogram was built based on the dis-similarity of the topological overlap, which presented 16 gene co-expression modules. The grey module indicates no co-expression between the genes. **B** Correlated heatmap plot of the adjacency modules in the WGCNA. The rectangle of each row and column represents a module eigengene. In the correlated heatmap plot, light blue represents low adjacency, whereas red represents high adjacency. WGCNA, weighted correlation network analysis.

The expression values of 12,604 genes detected in all 21 samples were used to construct the co-expression module using a gene hierarchical cluster network tool, R package, WGCNA (V1.69) (Peter et al., 2008). The correlation matrix and the adjacency matrix of the gene expression profile of the PEDV (GDS01 or HX)-infected samples were analyzed through the standard parameters of WGCNA. The co-expression modules were constructed and the hierarchical average linkage clustering method was utilized to identify the gene modules of each gene network. All samples were clustered, and a total of 16 different module eigengenes were observed based on their gene expression level (Fig. 3A). The total number of genes in all 16 modules is shown in Table 1. Interactions of the 16 co-expression modules were also analyzed and were depicted as a heatmap (Fig. 3B).

**Table 1:** Numbers of gene in each co-expression module.

Module	0	1	2	3	4	5	6	7	8	9	10	11	12	13	14	15	16
Number	124	3334	2385	1538	1212	1182	523	424	413	395	324	213	207	138	94	56	42

### 3.4 Gene expression trend analysis

**Fig. 4:**
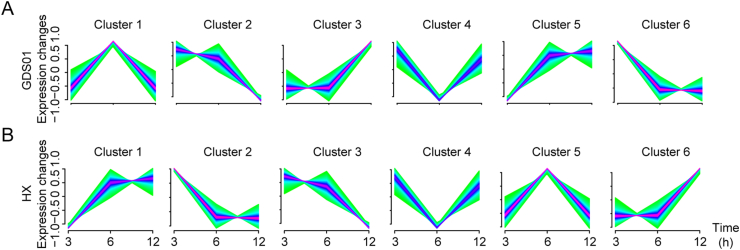
Graphs of six clusters in GDS01 or HX-infected cells along time series based on FPKM value of genes. **A** Clusters of genes upon GDS01 strain infection at 3, 6, and 12 hpi. **B** Clusters of genes upon HX infection at 3, 6, and 12 hpi. FPKM, fragments per kilobase per million; hpi, hours post infection.

To determine how IPEC-J2 cells respond to the PEDV infection, 16,942 and 16,000 genes identified in the HX and the GDS01 infection group, respectively, were classified into a total 12 of clusters (six clusters to each infection group) using the Mfuzz package (V2.48.0) (Lokesh et al., 2007) by considering their expression tendencies along with the time (Fig. 4A and B). In the GDS01 group, the genes classified in clusters 1, 2, and 5 were down-regulated, whereas the genes in clusters 3, 4, and 6 demonstrated up-regulation. Similarly, in the HX groups, clusters 1, 4, and 6 presented up-regulated genes, and clusters 2, 3, and 5 showed down-regulated genes. Those genes that clustered together were more likely considered to be classified into the same functional gene set.

### 3.5 GO enrichment analysis of DEGs

**Fig. 5:**
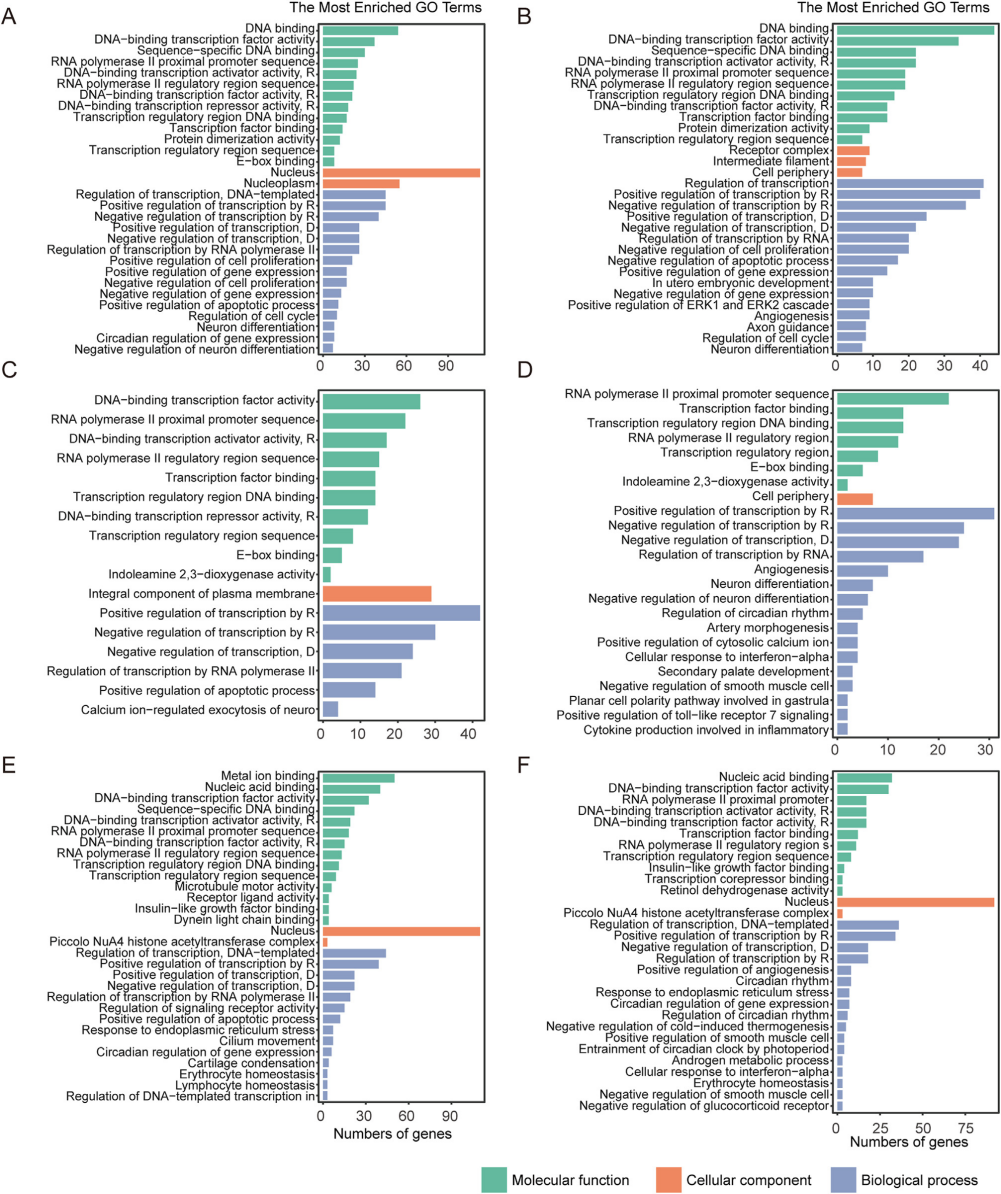
GO enrichment analysis of DEGs in IPEC-J2 cells in response to PEDV infection. **A, C, E** GO enrichment analysis of DEGs in the GDS01-infected groups at 3, 6, and 12 hpi; **B, D, F** GO enrichment analysis of DEGs in the HX-infected groups at 3, 6, and 12 hpi. PEDV, porcine epidemic diarrhea virus; GO, gene oncology; DEG, differentially expressed gene; hpi, hours post infection.

To know the biological importance of the DEGs, GO term enrichment analysis was performed by considering three functional categories that included molecular function, cellular components, and biological processes. For the stringency purpose, the top 30 or less highly enriched GO terms (Q-value < 0.05) corresponding to all DEGs were considered (Fig. 5). At each infection time-point, the GDS01 and HX groups shared a similar GO enrichment pattern, but the number of DEGs enriched in GDS01 group was more than that in HX group. The genes enriched in the molecular function category are mainly associated with DNA-binding and DNA-binding transcription factor activity. The genes enriched in cellular component were markedly less than the other two GO categories, and were predicted to localize primarily in the nucleus, cell periphery, and intermediate filament. The genes relevant to nuclear distribution were enriched at 3 and 12 hpi in the GDS01 group but only at 12 hpi in the HX group. The biological process classification predicted the enrichment of DEGs mainly in the regulation of cellular transcription, RNA polymerase II activity, cellular proliferation, and apoptosis.

### 3.6 Pathways enrichment analysis of DEGs

**Fig. 6:**
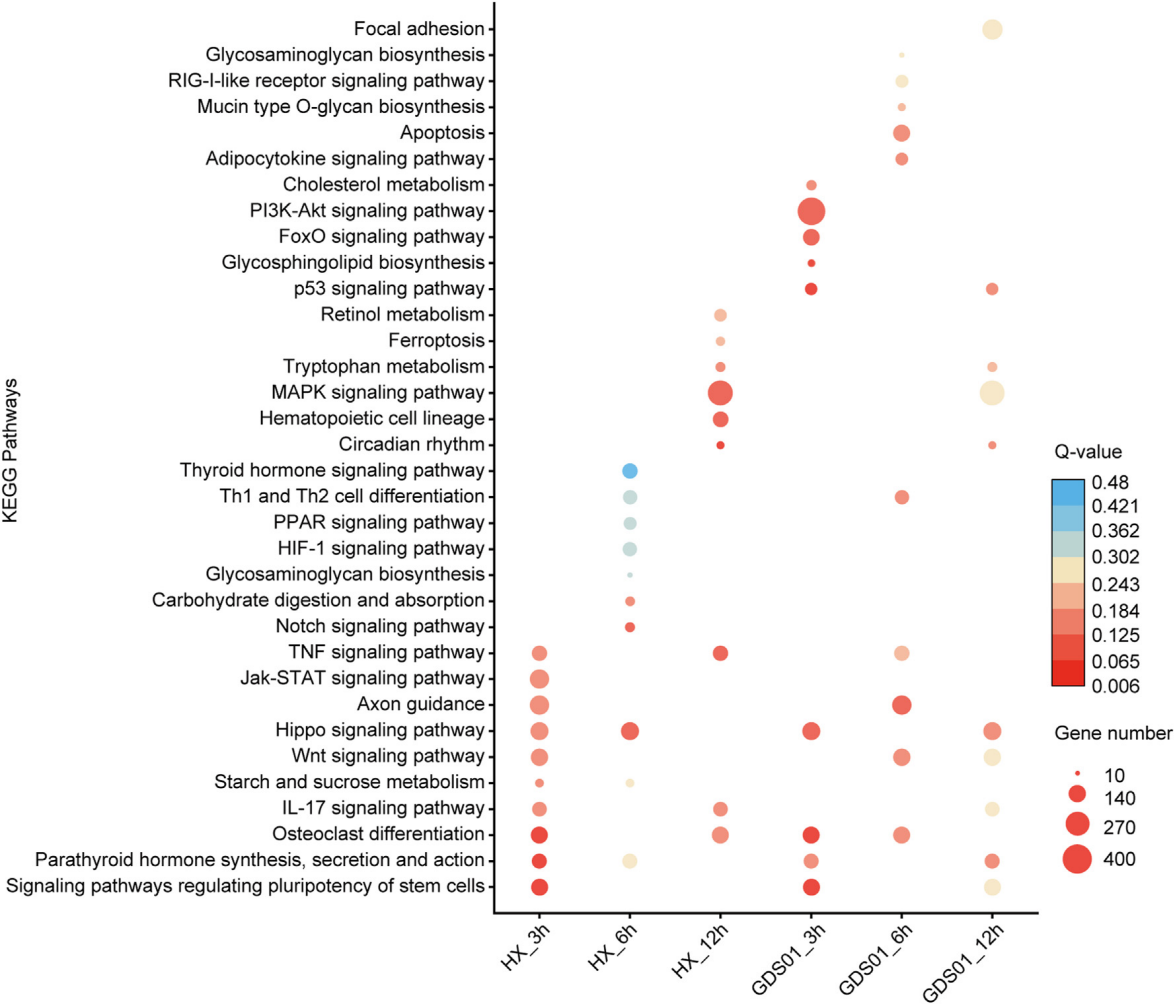
Dot map showing the compilation of top 10 enriched KEGG pathways in all PEDV infection groups. Each vertical column of the dot represents a PEDV infection group. PEDV, porcine epidemic diarrhea virus; KEGG, Kyoto encyclopedia of genes and genomes.

To identify the cellular pathways potentially involved during the PEDV infection, the KEGG database was employed. The top 10 highly enriched biological pathways corresponding to each condition in both infection groups are shown together in a bubble diagram (Fig. 6). Upon infection with the HX strain, the KEGG pathways were observed to be enriched in the immune-associated pathways such as TNF and IL-17 signaling. In contrast, the GDS01 infection resulted in marked induction of apoptosis- and autophagy-related pathways, including PI3K-Akt, p53, and FoxO signaling. The most commonly enriched pathways among all the infection conditions are Hippo signaling and osteoclast differentiation. In addition, the highest number of DEGs were found to associate with the PI3K-Akt signaling in the GDS01 group and with the MAPK signaling in the HX group.

**Fig. 7:**
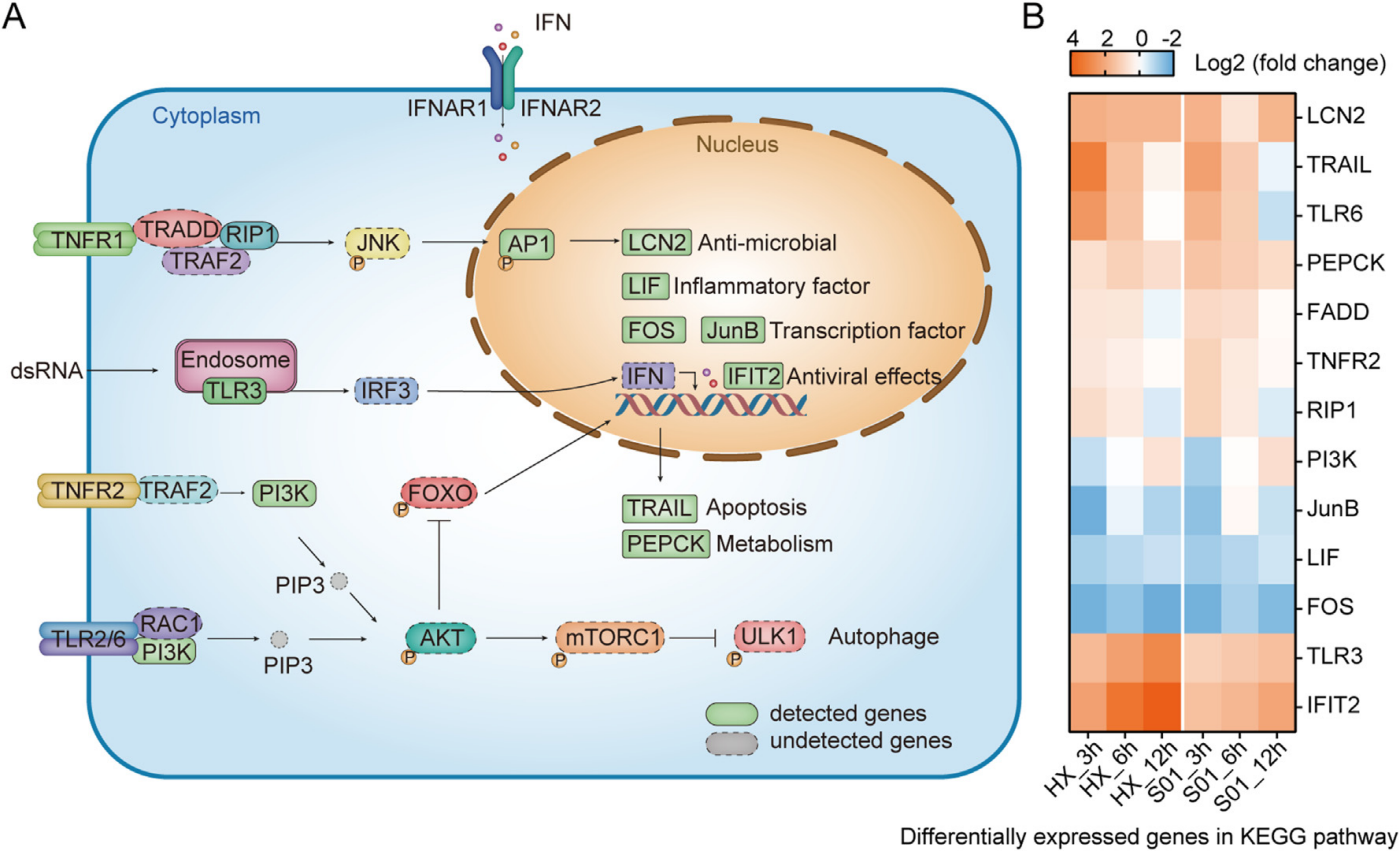
Signaling pathways affected by PEDV infection. **A** Schematic representation of the signaling pathways that converge on immune response, apoptosis, autophagy and metabolism. The proteins detected in PEDV-infected cells are shown in the solid boxes, whereas the proteins that remained undetected in our study are shown in the dotted boxes. **B** Heatmap shows the fold change of detected genes in PEDV-infected cells compared with mock-infected cells at 3, 6 and 12 hpi. PEDV, porcine epidemic diarrhea virus; hpt, hours post-infection.

In order to understand the host immune defense response upon PEDV infection, the four most enriched immune signaling pathways are displayed in Fig. 7A. The DEGs identified in our study are shown in the solid boxes, and their fold change values normalized to the value of their negative control are presented in the form of a heatmap (Fig. 7B). The up-regulation of TLR3, PI3K and the down-regulation of TRAIL, and RIP1 were noticed in a time-dependent manner in both infection groups. Two DEGs exhibited a time-specific expression pattern in both infection groups: reduction of FADD at 12 hpi and elevation of JunB at 6 hpi. Moreover, LCN2/PEPCK/TNFR2 and LIF/FOS showed almost constant up- and down-regulated expression, respectively, across all three infection time-points studied in both groups.

Overall, these findings predict that both PEDV strains GDS01 and HX may induce the alteration of the host cellular pathways to facilitate their own replication.

### 3.7 Confirmation of DEGs by quantitative real-time PCR

**Fig. 8:**
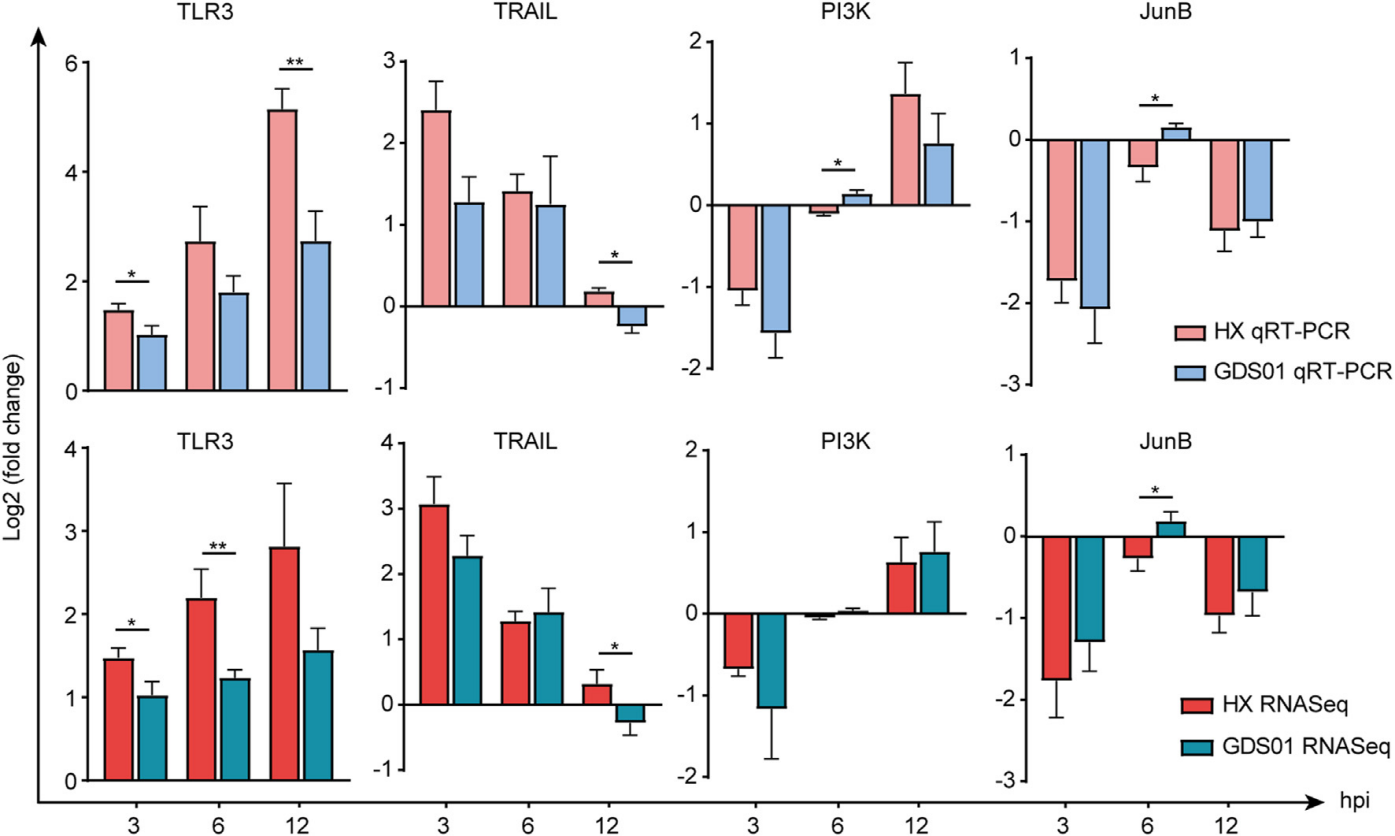
Validation of RNA-sequencing data by quantitative real-time PCR. Cultured IPEC-J2 cells were mock-infected or infected with PEDV strain GDS01 or HX at MOI of 1. At 3, 6, and 12 hpi, samples were harvested and relative mRNA expression levels of indicated genes were measured by quantitative real-time PCR. Data are expressed as mean ​± ​SEM from three independent experiments. Data were analyzed using the Mann-Whitney test. ∗*P* ​< ​0.05, ∗∗*P* ​< ​0.01. PEDV, porcine epidemic diarrhea virus; MOI, multiplicity of infection; SEM, standard error of mean; hpi, hours post infection.

To confirm the differential expression of the PEDV-induced genes detected in our sequencing data, we chose four DEGs (TLR3, TRAIL, PI3K and JunB) as predicted above to be engaged in immune response, apoptosis, and autophagy, and then validated their expression in PEDV (GDS01 or HX)-infected or mock-infected IPEC-J2 cells by quantitative real-time PCR. Primers used in our study are listed in Table 2. As shown in Fig. 8, these DEGs exhibited similar expression signatures as were detected in the sequencing data, indicating the robustness of our experimental settings and bioinformatic analyses. When compared to the gene expression pattern upon HX infection, TLR3 was observed to show an upregulation pattern at three infection time-point, TRAIL and TLR6 genes exhibited a downregulated pattern at 12 ​h infection time-point, whereas PI3K and JunB showed an upregulated pattern at 6 ​h infection time-point, upon GDS01 infection. Overall, these results validate our sequencing data and highlight some viral strain-dependent gene expression patterns.

**Table 2:** Quantitative real-time PCR primers used in this study.

Genes symbol	Forward primer	Reverse primer
GAPDH	CGATGGTGAAGGTCGGAGTGAACGG	CCTGGAAGATGGTGATGGGATTTCC
TLR3	TCTCGAAGCAGTTTGAAACAC	GCGCCGCTCAATCTTATTGG
TRAIL	TAATTGGCTAAATGATCTGC	GCCTTAACCTATTGGCTCT
JunB	AGCCCATCCCCGCTGTCCATAAAG	CAGGGTCAACTGTACAGGCATCTT
PI3K	GTGACTGACTGTGTAAGCCCA	GACTGGCACCTAGAACGTGA
IFIT2	AAGCACCTCAAAGGGCAAAAC	TCGGCCCATGTGATAGTAGAC

### 3.8 TLR3 restricts the replication of HX strain by enhancing the antiviral *IFIT2* gene expression

**Fig. 9:**
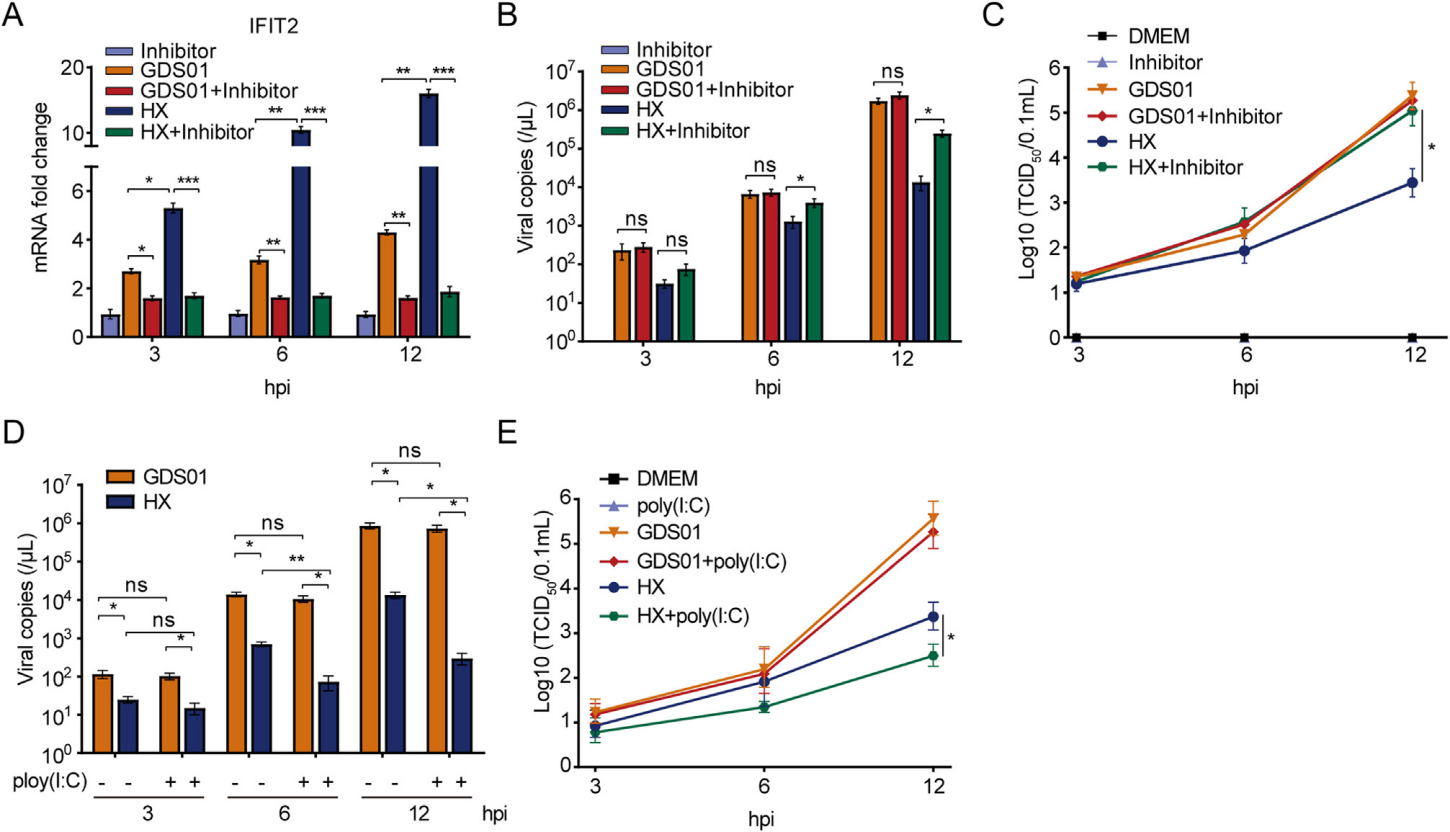
Inhibition of TLR3 reduces IFIT2 expression and facilitates the HX strain replication. **A** IFIT2 mRNA expression level measured by quantitative real-time PCR. **B** Viral copies in the culture supernatants detected through the quantitative real-time PCR. **C** Viral titers in the culture supernatants determined by TCID_50_ assay. **D** Viral copies in culture supernatants detected through the quantitative real-time PCR with poly(I:C) stimulation. **E** Viral titers in the culture supernatants determined by TCID_50_ assay with poly(I:C) stimulation. Data are expressed as mean ​± ​SEM from three independent experiments. Data were analyzed using the Mann-Whitney test. ∗*P* ​< ​0.05, ∗∗*P* ​< ​0.01, ∗∗∗*P* ​< ​0.001. ns, non-significant; PCR, polymerase chain reaction; TCID_50_, the 50% tissue culture infectious dose; SEM, standard error of mean.

To discern a possible role of detected genes in regulating the PEDV replication, we selected TLR3 as one of the DEGs in the sequencing data. In the HX-infected IPEC-J2 cells, the TLR3 expression was observed to be significantly increased than that in the GDS01-infected cells, which might have contributed to the lower propagation of HX strain when compared to GDS01 strain (Fig. 7B). To verify this speculation, we employed a selective TLR3 inhibitor in a series of experiments and examined its effect on the downstream antiviral *IFIT2* gene and PEDV (HX and GDS01) replication in IPEC-J2 cultured cells. It was observed that TLR3 inhibitor caused a reduction of IFIT2 mRNA expression level (Fig. 9A). Furthermore, the reduced expression of IFIT2 mRNA upon TLR3 inhibition was associated with significantly enhanced viral copies and titers of HX strain, as measured by the quantitative real-time PCR and TCID_50_ assay, respectively (Fig. 9B and C). No significant effect of TLR3 inhibition was noticed on the viral copies and titers of GDS01 strain (Fig. 9B and C). We then examined the effect of poly(I:C) treatment on the replication of both viral strains. It was observed that the replication of HX strain in IPEC cells was significantly suppressed upon poly(I:C) stimulation, whereas the replication of GDS01 strain was impervious to poly(I:C) transfection, as determined by the quantitative real-time PCR and TCID_50_ assay (Fig. 9D and E). Hence, these data suggest a GDS01-specific immune escape mechanism that facilitates the GDS01 strain replication.

**Fig. 10:**
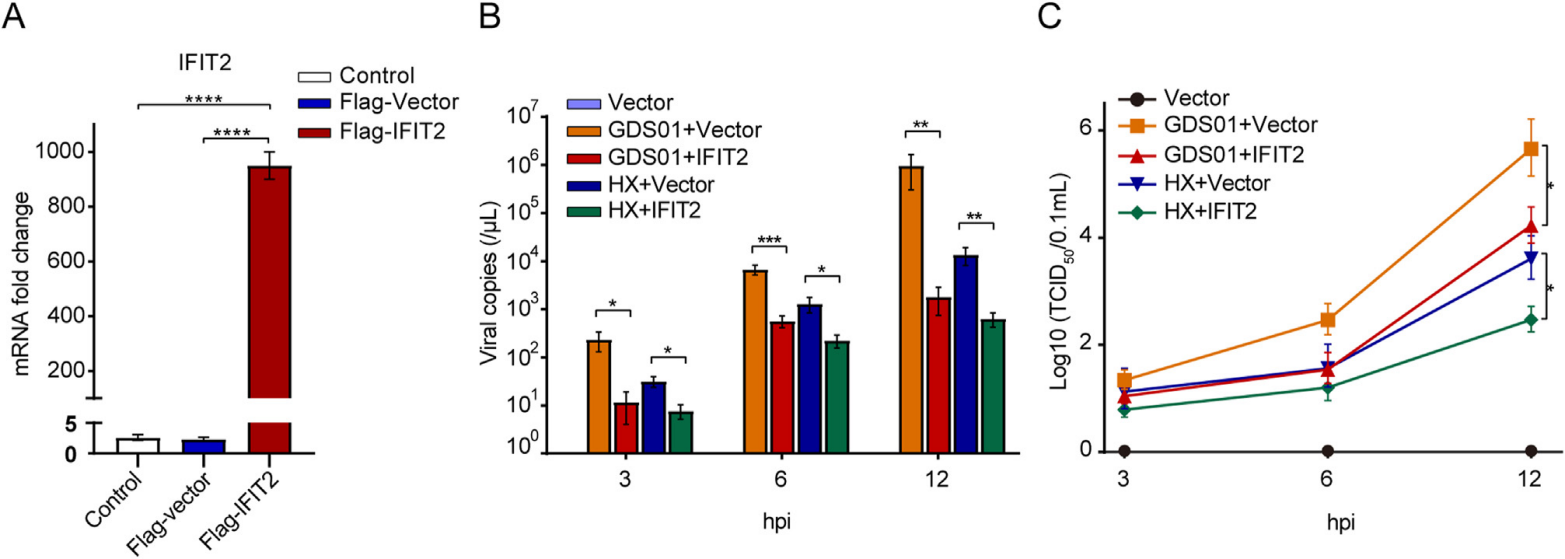
TLR3 induced antiviral *IFIT2* gene expression restricts the HX strain replication. **A** IFIT2 mRNA expression levels measured by quantitative real-time PCR. **B** Viral copies in the culture supernatants detected through the quantitative real-time PCR. **C** Viral titers in the culture supernatants determined by TCID_50_ assay. Data are expressed as mean ​± ​SEM from three independent experiments. Data were analyzed using the Mann-Whitney test. ∗*P* ​< ​0.05, ∗∗*P* ​< ​0.01, ∗∗∗*P* ​< ​0.001, ∗∗∗∗*P* ​< ​0.0001. PCR, polymerase chain reaction; TCID_50_, the 50% tissue culture infectious dose; SEM, standard error of mean.

We next confirmed the role of the *IFIT2* genes in the TLR3-mediated inhibition of HX strain. To this end, cultured IPEC-J2 cells were transfected with empty vector or Flag-IFIT2 plasmids, followed by infection with HX or GDS01 strain for the indicated time points. The efficient transcription of IFIT2 mRNAs in transfected cells was validated by quantitative real-time PCR (Fig. 10A). Overexpression of IFIT2 caused a marked suppression of viral copies and titers of both HX and GDS01 strains, determined by quantitative real-time PCR and TCID_50_ assay, respectively (Fig. 10A and B).

Taken together, these data indicate that TLR3, via augmenting the antiviral IFIT2 gene expression, restricts the HX strain replication.

## 4 Discussion

Advancements in RNA-sequencing technologies have provided us an opportunity to understand the complex host-virus interaction in detail (Jones et al., 2014; Liu et al., 2019). Previous studies have performed the global analysis of host transcriptomic signatures to gain insights into the host response to PEDV infection at the late stages of virus infection (Hu et al., 2020; Shen et al., 2020). Herein, we provide, for the first time, a view of genome-wide alterations in the host transcriptome that occur in response to PEDV infection, with a focus on differences in the host response to virulent (GDS01) and avirulent (HX) PEDV strains at the early stages of infection. Upon RNA-sequencing analysis of cultured IPEC-J2 cells infected with either GDS01 or HX viruses, we detected a total of 1968 significant DEGs in both infection groups (GDS01 group: 1065 genes and HX group: 903 genes), which were predicted to regulate mainly cellular transcription, antiviral immune response, autophagy, and apoptosis. The findings obtained from this study highlighted the intricate regulation of cellular genes during the initial phases of PEDV infection, and suggested their potential role in regulating the PEDV pathogenesis. Further studies are required to understand the biological impact of identified DEGs during PEDV infection. Whether or not, targeting these genes confers a therapeutic effect against PEDV infection, remains to be evaluated in future studies.

The differences in the gene expression pattern observed upon infection with virulent and avirulent PEDV strains could pave a way to better understand the strain-specific host responses, which could be considered for developing the novel antiviral strategies. Previous studies mainly examined the transcriptomic profile of pig enterocytes infected with a classical avirulent PEDV CV777 strain (Hu et al., 2020) or Vero E6 cells infected with mutated virulent JS201603 strain (Zhang et al., 2018a). In contrast, we examined and compared the transcriptomic landscape of pig enterocytes upon infection with a virulent and an avirulent PEDV strains. The comparative analysis of host response to both viral strains revealed that the overall modulation of genes enriched in immunity/inflammation was stronger upon infection with avirulent HX strain, whereas the virulent GDS01 strain intensified the response of autophagy and apoptosis-associated genes, thus uncovering the potential mechanisms of virulence associated with the GDS01 strain. In a recent study, Hu et al. found that the DEGs detected in CV777-infected IPEC-J2 cells were enriched to the inflammatory response (GO:0006954), immune response (GO:0006955), regulation of inflammatory response (GO:0050727), innate immune response (GO:0045087) categories (Hu et al., 2020), which was in agreement with our GO term enrichment analysis of DEGs in the avirulent HX group. It would be intriguing to perform the extensive functional analysis of these detected genes in orchestrating the immunity to PEDV infection.

Autophagy has been shown to facilitate the replication of a virulent PEDV CH/YNKM-8/2013 stain at the late stages of infection but not at the early stages, via engaging the autophagy regulators and RNA interference in Vero cells (Guo et al., 2017). The quantitative proteomic analysis of PEDV CV777 vaccine strain-infected Vero cells at 48 hpi indicated that the differentially expressed proteins were highly enriched in the autophagy pathway (Sun et al., 2015). These results are inconsistent with our finding of strong enrichment of genes in the autophagy pathway at the early stages of virulent GDS01 strain infection in IPEC-J2 cells. Such observations might be associated with the infection stage, strain, and cell-type specificity, which required additional investigations. Furthermore, the proteomic analysis is required to confirm, whether or not, the dysregulated proteins during the early stage of GDS01 strain infection are enriched in the autophagy pathway.

PEDV has been shown to induce apoptosis *in vitro* and *in vivo*, which plays an important role in cytotoxicity and pathogenesis (Kim et al., 2014; Xu et al., 2019; Zeng et al., 2015). For instance, the PEDV virulent SM98-1 strain infection promoted the nuclear translocation of mitochondrial apoptosis-inducing factor, which subsequently enhanced the virus replication in cultured Vero cells and enterocytes of experimentally-infected piglets by inducing apoptosis in a caspase-independent manner (Oh et al., 2020). In a similar context, the S1 spike protein of virulent SM98-1 strain caused apoptosis of Vero cells, which was found to be associated with the cleavage of apoptosis-inducing factor mitochondria associated-1 and poly (ADP ribose) polymerase-1 and with the activation of caspase-3 and caspase-8 (Chen et al., 2018). These findings support our observation of strong enrichment of DEGs in apoptotic pathway during infection of IPEC-J2 cells with the virulent GDS01 strain. Since little is known about the mechanisms by which PEDV induces apoptosis, the genes and pathways identified to be involved in the apoptosis in our study, could be utilized to uncover the precise mechanisms of PEDV-induced apoptosis.

PEDV evades the host immunity by two distinct mechanisms: 1) protecting the viral genome from the cellular nucleic acid sensors and 2) producing the virus-encoding interferon antagonists (Li et al., 2020). PEDV nonstructural protein 1, 3, 5, 7, 8, 14, and 15 blocks the type I and/or type III interferon production by inhibiting the nuclear translocation of NF-κB and IRF1, cleaving NEMO, deubiquitinating RIG-I and STING, or through some other unknown mechanisms (Li et al., 2020; Zhang et al., 2017, 2018b). PEDV AJ1102 strain nucleocapsid protein sequesters the IRF3-TBK1 interaction, which leads to the suppressed activity of NF-κB and the reduced synthesis of type I interferon (Ding et al., 2014). The difference in the activation or suppression of immune signaling pathways has also been associated with the strain-type, time of infection, or cell-specificity (Du et al., 2019). For example, PEDV virulent non-S-INDEL strain impedes the NF-κB signaling pathway and reduces subsequent cytokine/interferon production by negatively regulating the TLR4, TLR7, TLR8, and TLR9 expression, whereas the less-virulent PEDV S-INDEL strain enhances the cytokines production through the non-canonical NF-κB pathway by stimulating RIG-I (Temeeyasen et al., 2018). In our study, we observed that the HX strain stimulated the TLR3 and the downstream antiviral IFIT2 gene expression to a relatively higher extent than GDS01 strain, which led to reduced HX strain, but not GDS01 strain, replication, highlighting an immune evasion mechanism that was specific to GDS01 strain. Additionally, we observed that the ectopic expression of IFIT2 in cells suppressed both HX and GDS01 replication. The reduced replication of GDS01 strain upon expression of IFIT2, but not TLR3, suggested the engagement of TLR3-independent antiviral mechanisms, which could be intriguing to examine in future studies. Moreover, the obvious differences in the secondary and tertiary RNA structures of both viral strains may contribute to a differential TLR3 recognition, thus causing a strain-specific response.

## 5 Conclusions

In summary, we performed the global gene expression profiles of pig enterocytes infected by virulent or avirulent PEDV strains. Upon integration of RNA-sequencing, functional enrichment analyses, gene co-expression network analysis, experimental validations, and functional studies, we highlighted similarities and differences in gene expression pattern and cellular processes/pathways perturbed at the early-stage infection of PEDV virulent and avirulent strains. This study may shed light on molecular mechanisms involved during the PEDV replication. As the transcriptomic signatures at the early stages of PEDV infection have not yet been explored, our study may serve as a valuable resource for PEDV-related research studies in the future.

## Data availability

The raw data of RNA-sequencing have been uploaded to National Center for Biotechnology Information (NCBI) (https://www.ncbi.nlm.nih.gov) and the accession numbers are from SRR14552484 to SRR14552504.

## Ethics statement

This article does not contain any studies with human or animal subjects performed by any of the authors.

## Author contributions

Ouyang Peng: conceptualization, software, formal analysis, resources, data curation, writing-original draft preparation, visualization. Xiaona Wei: conceptualization, methodology, investigation, resources. Usama Ashraf: methodology, writing-review and editing. Fangyu Hu: formal analysis, resources, data curation. Yongbo Xia: investigation. Qiuping Xu: writing-review and editing. Guangli Hu: investigation. Chunyi Xue: validation, writing-review and editing, supervision. Yongchang Cao: validation, supervision, funding acquisition. Hao Zhang: conceptualization, methodology, formal analysis, resources, project administration, funding acquisition.

## Conflict of interest

We declare that we do not have any commercial or associative interest that represents a conflict of interest in connection with the work submitted.

## Appendix A Supplementary data

Supplementary data 1 Supplementary data 1

Supplementary data 2 DEGs__KEGG_GO_Cluster Supplementary data 2 DEGs__KEGG_GO_Cluster

Supplementary Fig. S1 Genes expression levels distribution. **A** Boxplots show the distribution of gene expression levels in each sample. **B** Venn diagrams show unique and overlapping DEGs among subgroups with the same virus strain infection at three infection time-points or among subgroups with different strains infection but the same infection time-point. Each circle represents a group of gene sets, and the areas superimposed by different circles represent the intersection of these gene sets. The non-overlapping part indicates the uniquely expressed genes, and the numbers indicate the number of genes in the corresponding area. DEG, differentially expressed gene. Supplementary Fig. S1

Supplementary Fig. S2 Volcano plots of DEGs detected in PEDV-infected IPEC-J2 cells. **A–C** Volcano plots of DEGs detected in the cells infected with GDS01 strain for a period of 3, 6, and 12 h. **D–F** Volcano plots of DEGs detected in the cells infected with HX strain for a period of 3, 6, and 12 h. The X-axis represents the fold change of the difference after conversion to log2 and the Y-axis represents the significance value after conversion to -log10. Red represents up-regulated DEGs, blue represents DEGs down-regulated DEGs, and gray represents non-DEGs. DEG, differentially expressed gene; PEDV, porcine epidemic diarrhea virus; NC, negative control. Supplementary Fig. S2

Supplementary Fig. S3 Diagram of pCMV-FLAG-IFIT2 overexpression plasmid. Supplementary Fig. S3
