# Supplementary material for: Genome-wide transcriptome analysis of porcine epidemic diarrhea virus virulent or avirulent strain-infected porcine small intestinal epithelial cells
Source: Virol Sin. 2022 Jan 18;37(1):70–81. doi: 10.1016/j.virs.2022.01.011 (PMC8922430; doi:10.1016/j.virs.2022.01.011)
Supplement: Supplementary data 1 [file mmc1.docx]

**Virologica Sinica**

**Supplementary Data**

**Genome-wide transcriptome analysis of porcine epidemic diarrhea virus virulent or avirulent strain-infected porcine small intestinal epithelial cells**

**Ouyang Peng^a^, Xiaona Wei^b^, Usama Ashraf^c^, Fangyu Hu^a^, Yongbo Xia^a^, Qiuping Xu^d^, Guangli Hu^a^, Chunyi Xue^a^, Yongchang Cao^a^ and Hao Zhang^a,*^**

*^a^ State Key Laboratory of Biocontrol, Life Sciences School, Sun Yat-sen University, Guangzhou, 510006, China*

*^b^ Wen’s Group Academy, Wen’s Foodstuffs Group Co, Ltd, Xinxing, Guangdong, 527400, China*

*^c^ State Key Laboratory of Agricultural Microbiology, Huazhong Agricultural University, Wuhan, 430070, China*

*^d^ Guangdong Provincial Key Laboratory of Malignant Tumor Epigenetics and Gene Regulation, Sun Yat-sen Memorial Hospital, Sun Yat-sen University, Guangzhou, 510120, China*

^*^ Corresponding author.

*E-mail addresses*: [zhanghao5@mail.sysu.edu.cn](mailto:zhanghao5@mail.sysu.edu.cn) (H. Zhang).

**
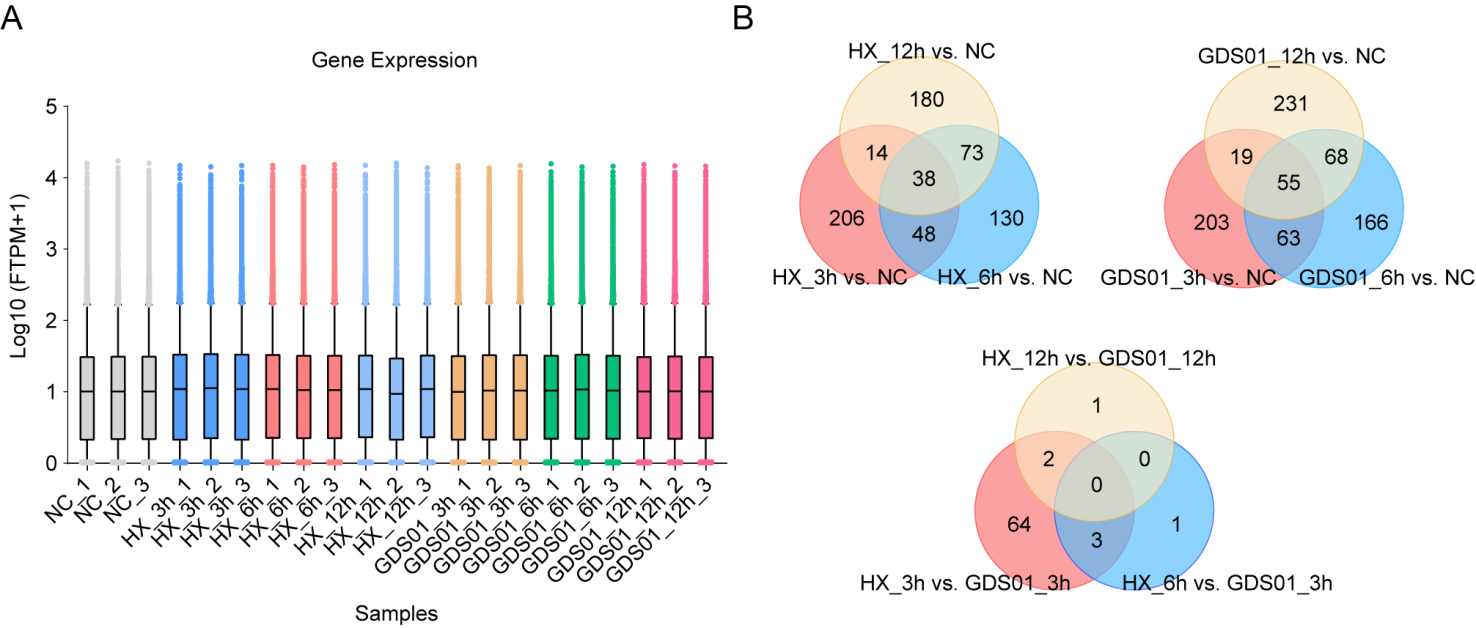
**

**Supplementary Fig. S1** Genes expression levels distribution. **A** Boxplots show the distribution of gene expression levels in each sample. **B** Venn diagrams show unique and overlapping DEGs among subgroups with the same virus strain infection at three infection time-points or among subgroups with different strains infection but the same infection time-point. Each circle represents a group of gene sets, and the areas superimposed by different circles represent the intersection of these gene sets. The non-overlapping part indicates the uniquely expressed genes, and the numbers indicate the number of genes in the corresponding area. DEG, differentially expressed gene.

**
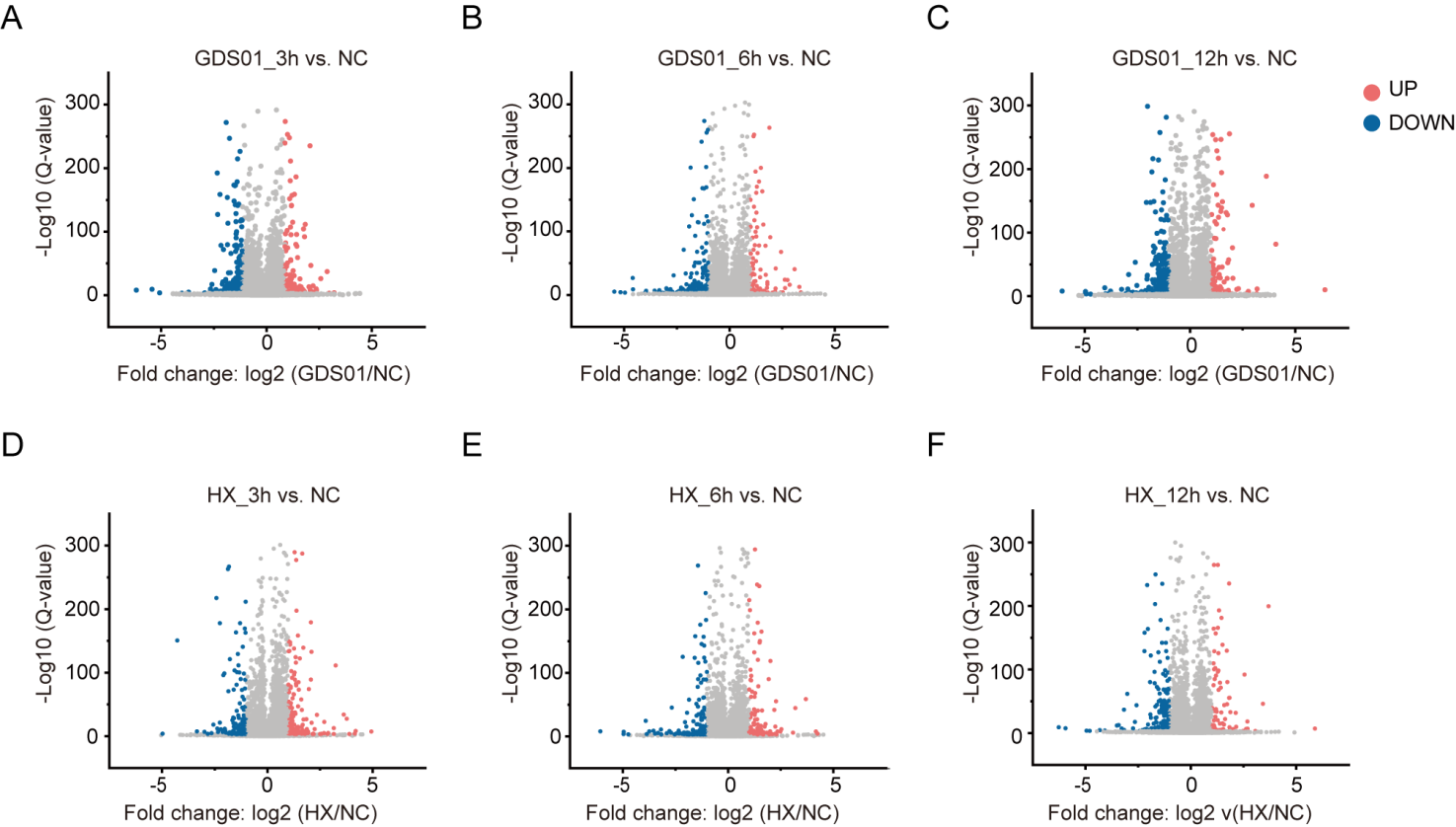
**

**Supplementary Fig. S2** Volcano plots of DEGs detected in PEDV-infected IPEC-J2 cells. **A**–**C** Volcano plots of DEGs detected in the cells infected with GDS01 strain for a period of 3, 6, and 12 h. **D**–**F** Volcano plots of DEGs detected in the cells infected with HX strain for a period of 3, 6, and 12 h. The X-axis represents the fold change of the difference after conversion to log2 and the Y-axis represents the significance value after conversion to -log10. Red represents up-regulated DEGs, blue represents DEGs down-regulated DEGs, and gray represents non-DEGs. DEG, differentially expressed gene; PEDV, porcine epidemic diarrhea virus; NC, negative control.

**
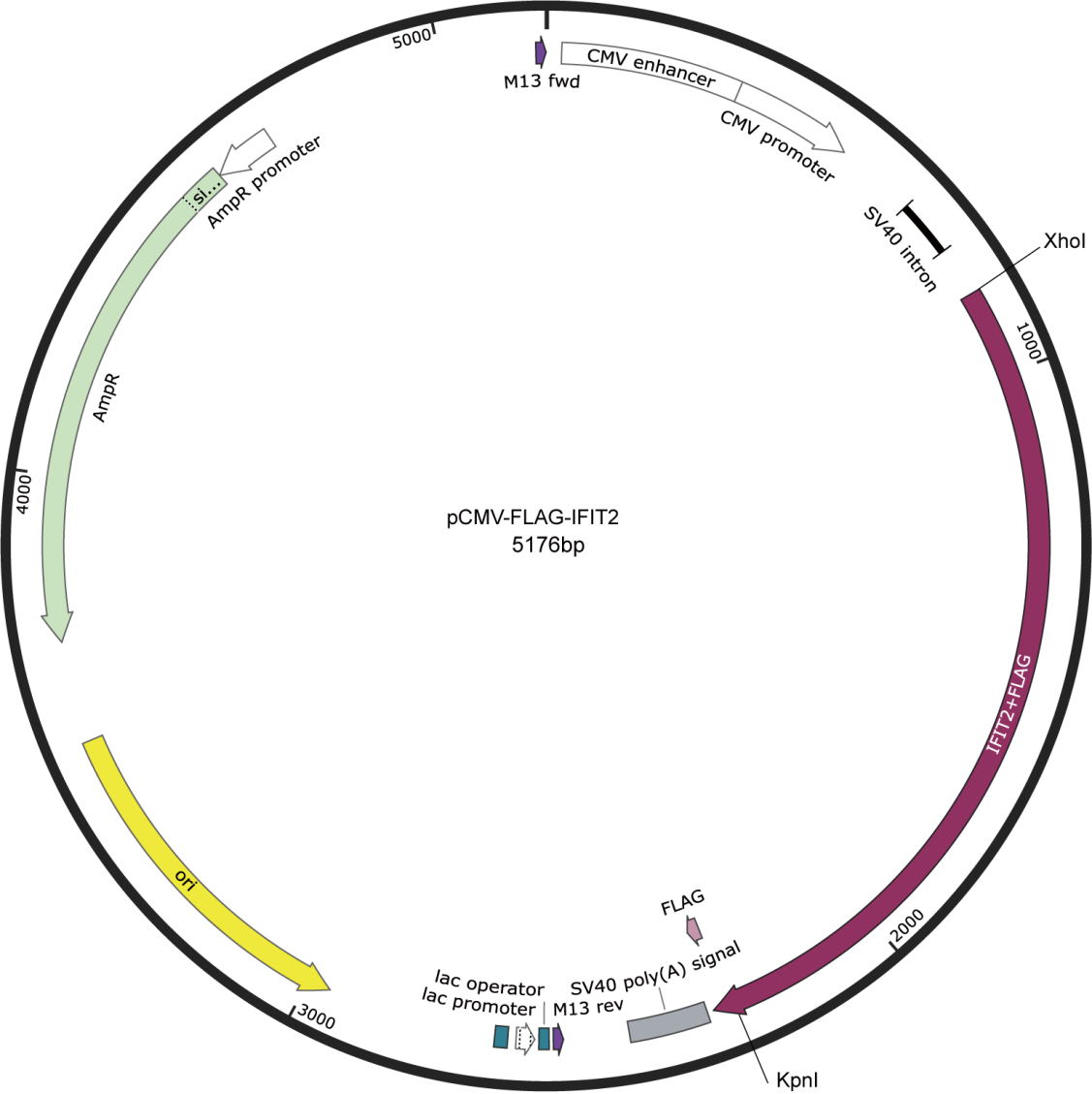
**

**Supplementary Fig. S3** Diagram of pCMV-FLAG-IFIT2 overexpression plasmid.
