# Supplementary figures and images for: Genome-wide transcriptome analysis of porcine epidemic diarrhea virus virulent or avirulent strain-infected porcine small intestinal epithelial cells
Source: Virol Sin. 2022 Jan 18;37(1):70–81. doi: 10.1016/j.virs.2022.01.011 (PMC8922430; doi:10.1016/j.virs.2022.01.011)

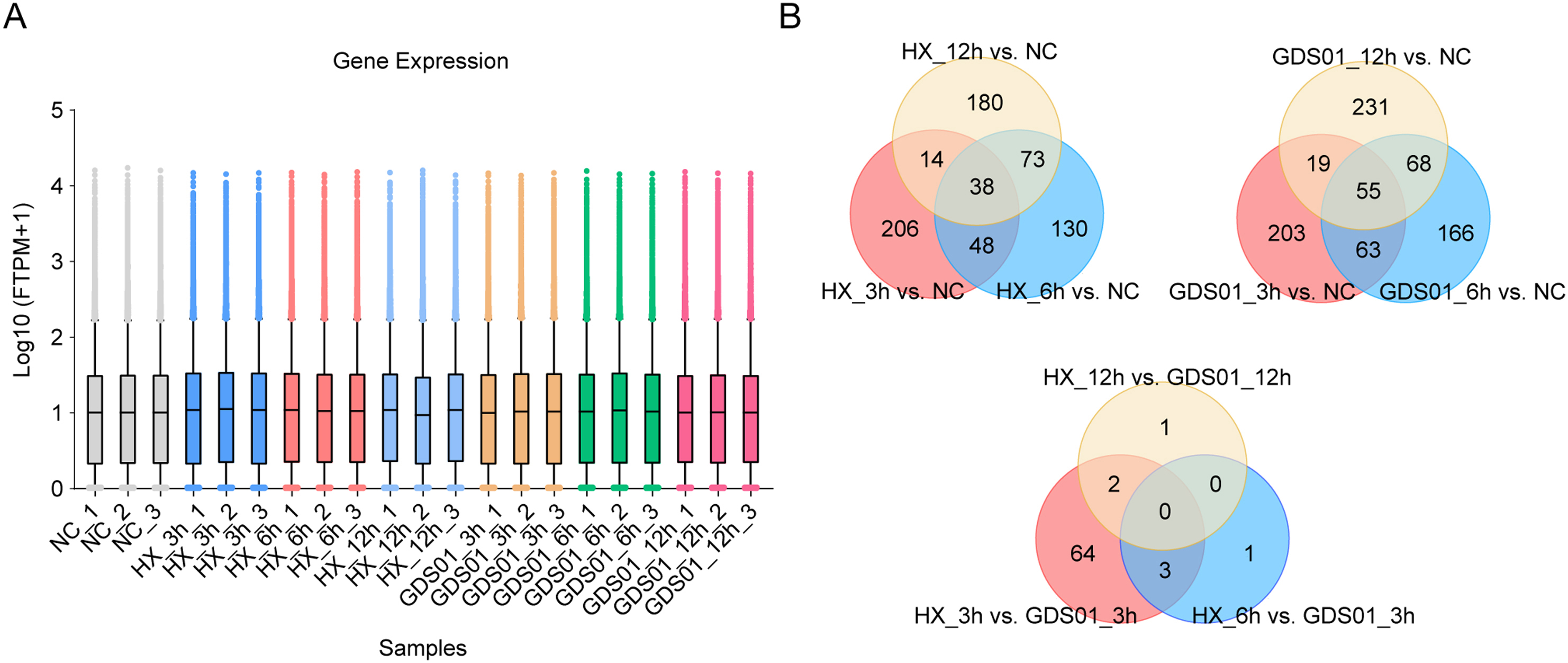

Supplement: Supplementary Fig. S1 — Genes expression levels distribution. A Boxplots show the distribution of gene expression levels in each sample. B Venn diagrams show unique and overlapping DEGs among subgroups with the same virus strain infection at three infection time-points or among subgroups with different strains infection but the same infection time-point. Each circle represents a group of gene sets, and the areas superimposed by different circles represent the intersection of these gene sets. The non-overlapping part indicates the uniquely expressed genes, and the numbers indicate the number of genes in the corresponding area. DEG, differentially expressed gene. [file figs1.jpg]

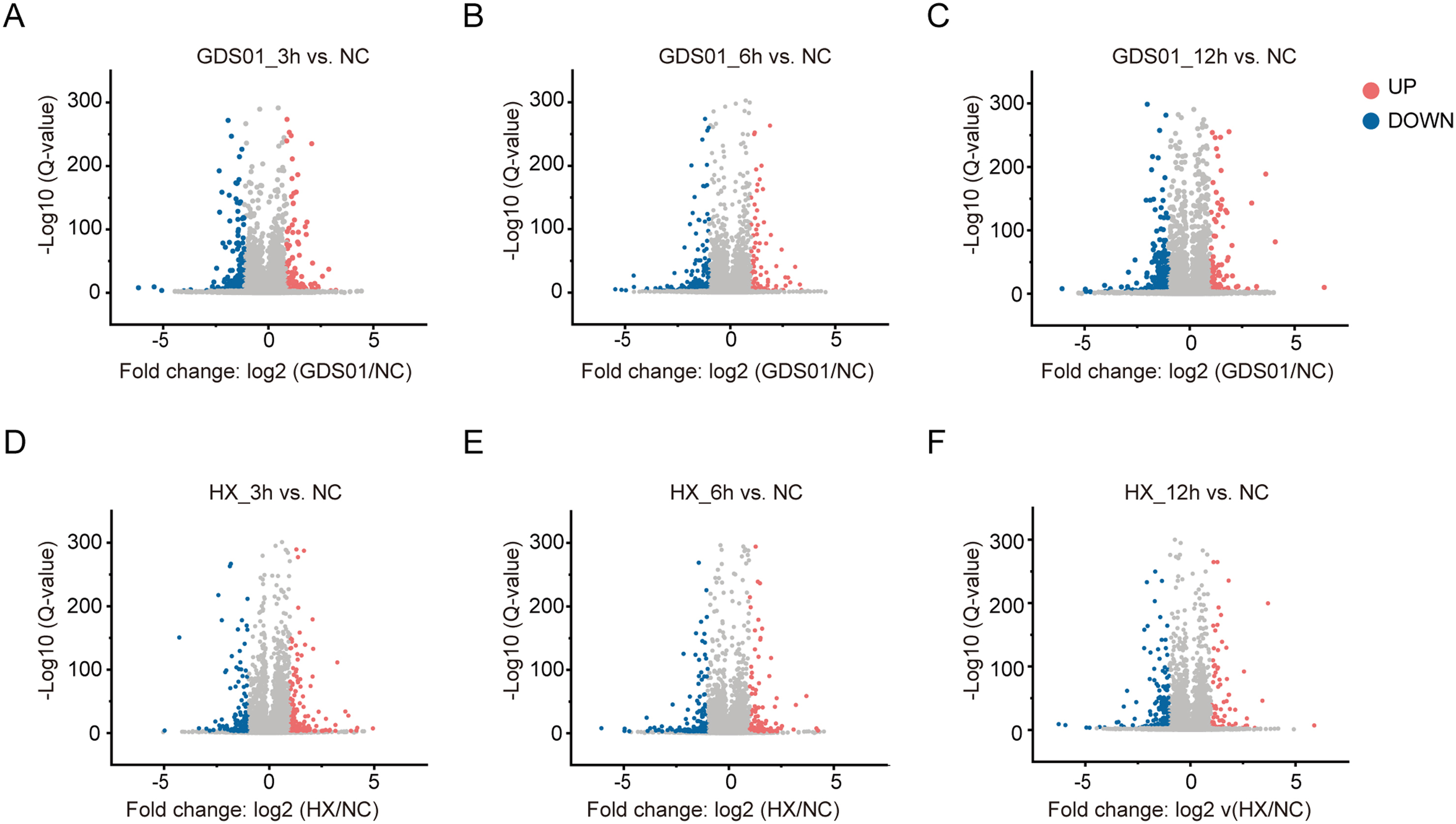

Supplement: Supplementary Fig. S2 — Volcano plots of DEGs detected in PEDV-infected IPEC-J2 cells. A–C Volcano plots of DEGs detected in the cells infected with GDS01 strain for a period of 3, 6, and 12 h. D–F Volcano plots of DEGs detected in the cells infected with HX strain for a period of 3, 6, and 12 h. The X-axis represents the fold change of the difference after conversion to log2 and the Y-axis represents the significance value after conversion to -log10. Red represents up-regulated DEGs, blue represents DEGs down-regulated DEGs, and gray represents non-DEGs. DEG, differentially expressed gene; PEDV, porcine epidemic diarrhea virus; NC, negative control. [file figs2.jpg]

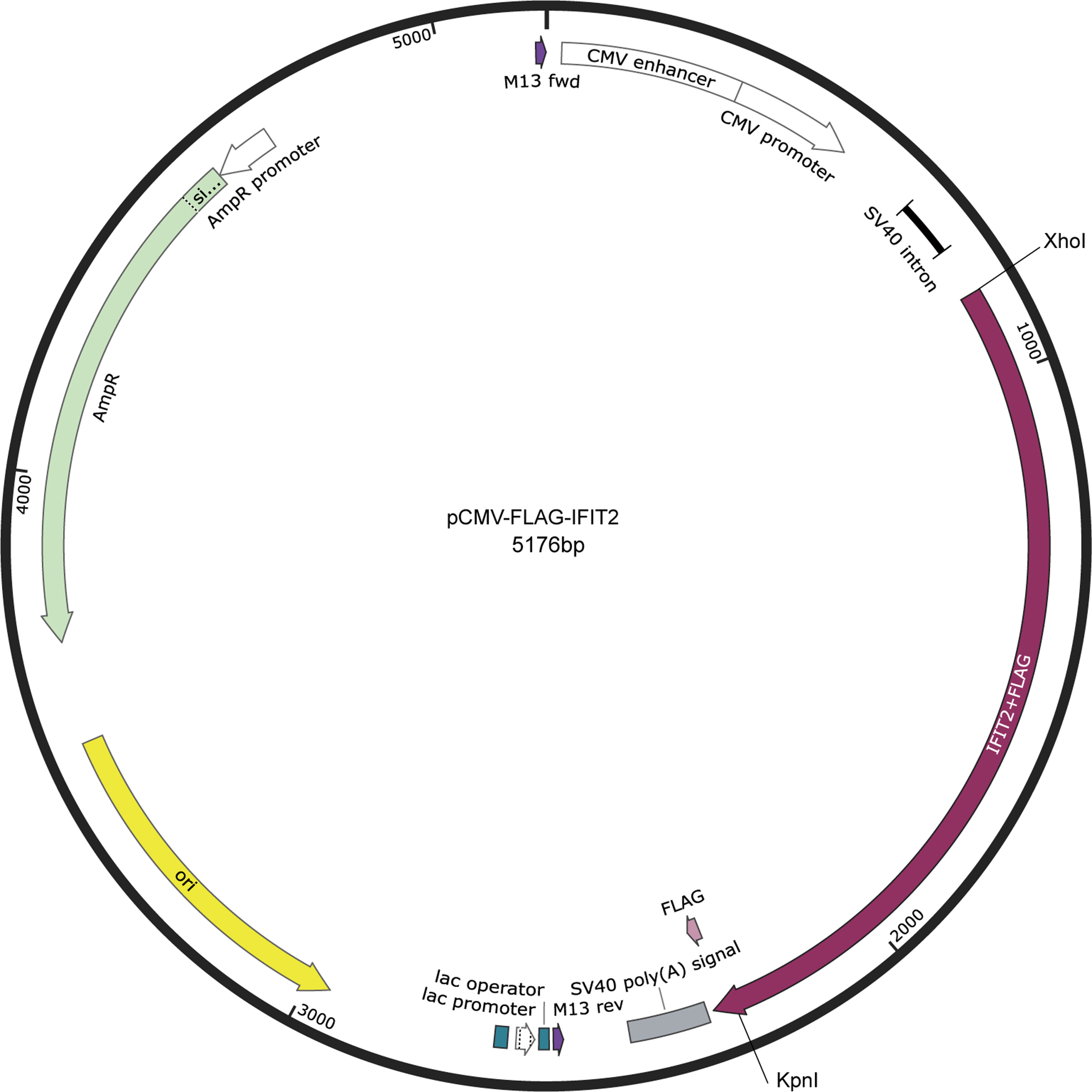

Supplement: Supplementary Fig. S3 — Diagram of pCMV-FLAG-IFIT2 overexpression plasmid. [file figs3.jpg]
